# Self-management dimensions associated with physical activity participation among gynecological cancer survivors: a cross-sectional study from the international InCHARGE consortium

**DOI:** 10.1186/s12905-026-04722-6

**Published:** 2026-07-30

**Authors:** Sindre Herskedal Fosstveit, Benedikte Western, Belle H de Rooij, Nicole P. M. Ezendam, Sveinung Berntsen, Ingrid Demmelmaier, Mette Skorstad, Mette Moustgaard Jeppesen, Stinne Holm Bergholdt, Pernille Tine Jensen, Ingvild Vistad

**Affiliations:** 1 Department of Sport Science and Physical Education, University of Agder, P.O. Box 422, Kristiansand, 4604 Norway; 2 Netherlands Comprehensive Cancer Organisation (IKNL), Utrecht, the Netherlands; 3 Center of Research on Psychological and Somatic Disorders (CoRPS), Department of Medical and Clinical Psychology, Tilburg University, Tilburg, the Netherlands; 4 Department of Public Health and Caring Sciences, Uppsala University, Uppsala, Sweden; 5 Department of Obstetrics and Gynecology, Sorlandet Hospital, Kristiansand, Norway; 6 Department of Gynaecology and Obstetrics, Lillebaelt University Hospital, Kolding, Denmark; 7 Odense Patient data Explorative Network, OPEN, Odense University Hospital, Odense, Denmark; 8 Department of Clinical Research, University of Southern Denmark, Odense, Denmark; 9 Department of Gynecology and Obstetrics, Dept of Clinical Medicine, Aarhus Universitetshospital, Aarhus University Hospital, Aarhus, Denmark; 10 Faculty of Medicine, University of Oslo, Oslo, Norway; 11 Research unit, Sorlandet Hospital, Kristiansand, Norway

**Keywords:** Cancer Survivors, Health Behavior, Motivation, Patient Participation

## Abstract

**Background:**

Physical activity (PA) participation is associated with improved outcomes among gynecological cancer survivors and can serve as an effective component of self-management. However, little is known about which self-management dimensions best support PA participation in gynecological cancer survivors. Therefore, the aim of the present study was to assess associations between different self-management dimensions and self-reported PA participation among gynecological cancer survivors.

**Methods:**

The analyses were conducted as part of the InCHARGE study, a multicenter, cross-sectional study of gynecological cancer survivors (*n* = 1433) in the Netherlands, Norway, and Denmark. Associations between seven self-management scales from the Health Education Impact Questionnaire (HeiQ) version 3 and PA as measured by the HeiQ Health Directed Activity scale were assessed. Unstandardized regression coefficients from the models adjusted for participant characteristics were reported.

**Results:**

Across endometrial, ovarian, and cervical cancer survivors, Positive and Active Engagement in Life (β = 0.66, 0.70, 0.74, respectively) and Self-Monitoring and Insight (β = 0.73, 0.61, 0.66, respectively) were associated with PA participation (*p* < 0.001).

**Conclusions:**

The present study findings indicate that self-management skills related to Positive and Active Engagement in Life and Self-Monitoring and Insight should be further assessed as intervention targets to improve PA levels in gynecological cancer survivors.

**Supplementary Information:**

The online version contains supplementary material available at https://doi.org/10.1186/s12905-026-04722-6.

## Background

Self-management has been suggested as a critical component of life beyond cancer [1]. Self-management support may function as an effective strategy to ensure that the long-term physical and psychological health needs of cancer survivors are addressed, especially as the population of cancer survivors is growing [1, 2]. Self-management involves managing the consequences of cancer and its treatment, understanding when and how to seek support, recognizing and reporting signs and symptoms of cancer recurrence, and implementing lifestyle changes to promote health, well-being, and survival [3]. Such lifestyle changes may include increased physical activity (PA) participation, which has been associated with better management of symptoms, side effects, and late effects during cancer treatment and recovery [4, 5]. Consequently, improving PA participation among cancer survivors has emerged as a topic of significant scientific interest [5].

Among endometrial, ovarian, and cervical cancer survivors, positive changes in PA have demonstrated associations with improved survival, general health, and quality of life, as well as decreased severity of comorbidities, depression, and anxiety [6–10]. However, a substantial proportion of gynecological cancer survivors are currently not physically active at a level meeting the recommended ≥ 150 min of moderate-to-vigorous intensity per week [11]. Insufficient PA levels two years post-diagnosis have been reported in 88%, 63%, and 45% of endometrial, cervical, and ovarian cancer survivors, respectively [10, 12, 13]. Consistent with this broader survivorship pattern, more than half of cancer survivors do not meet PA guidelines in large population-based surveillance data [14]. Despite extensive research on the effects of exercise and PA on health and well-being among cancer survivors, there is a notable gap in the literature regarding studies that specifically explore which components to target in order to improve PA behaviors in cancer populations [15–17]. Endometrial, ovarian, and cervical cancer are among the most commonly diagnosed gynecological malignancies, and improvements in early detection and treatment have contributed to a growing population of women living for many years beyond their diagnosis [18, 19]. The comparatively low PA levels in this population likely reflect population-specific characteristics, including a high prevalence of obesity, fatigue, functional limitations, and a considerable comorbidity burden, which together constitute disease-specific barriers to PA [13, 20, 21].

Self-management support and behavioral change may be imperative to achieve improved PA levels [22–25]. However, there is considerable content heterogeneity among self-management interventions, and the reporting of this content varies widely [1]. This variability poses challenges in identifying and establishing effective and feasible approaches to PA behavioral change [1, 26]. Furthermore, gynecological cancer survivors have received comparatively less attention in the literature compared to survivors with more prevalent cancer types, such as breast cancer, regarding effective means to improve PA behavior [20, 24, 27]. For example, in a systematic review of 131 randomized exercise oncology trials, breast cancer was the most frequently studied diagnosis (50% of trials), underscoring the predominance of breast cancer–focused intervention evidence [28]. As a result, evidence on effective targets for promoting PA in gynecological cancer survivors remains comparatively limited. Together with the population-specific barriers to PA outlined above, this limited evidence base makes it particularly important to identify modifiable self-management skills that support PA in gynecological cancer survivors. To strengthen the conceptual basis for identifying actionable targets, Self-Determination Theory suggests that sustained health behavior is more likely when autonomy, competence, and relatedness are supported [29]. Accordingly, self-management domains such as active engagement, self-monitoring/insight, and social support may be relevant for understanding PA participation. In Self-Determination Theory, autonomy refers to acting in accordance with one’s own values and interests, competence to feeling effective in achieving desired outcomes, and relatedness to feeling connected to and supported by others [29]. Within the Health Education Impact Questionnaire (HeiQ), these constructs correspond most closely to Positive and Active Engagement in Life (autonomous, value-driven engagement), Self-Monitoring and Insight and Skill and Technique Acquisition (self-regulatory competence), and Social Integration and Support (relatedness). Psychological factors, including emotional regulation and the way survivors process information and make decisions about their care, have also been identified as relevant targets for supportive interventions in cancer survivorship [30, 31]. To address these gaps, it is important to determine which modifiable self-management skills are associated with higher PA in gynecological cancer survivors, as this can help identify candidate targets to be evaluated in future intervention studies. Therefore, the objective of the present study was to assess the association between different dimensions of self-management and self-reported PA participation among gynecological cancer survivors. Guided by Self-Determination Theory, we hypothesized that self-management domains aligned with autonomous engagement and self-regulatory competence would be positively associated with PA participation among gynecological cancer survivors [29, 32, 33].

## Methods

### Design and participants

**Fig. 1:**
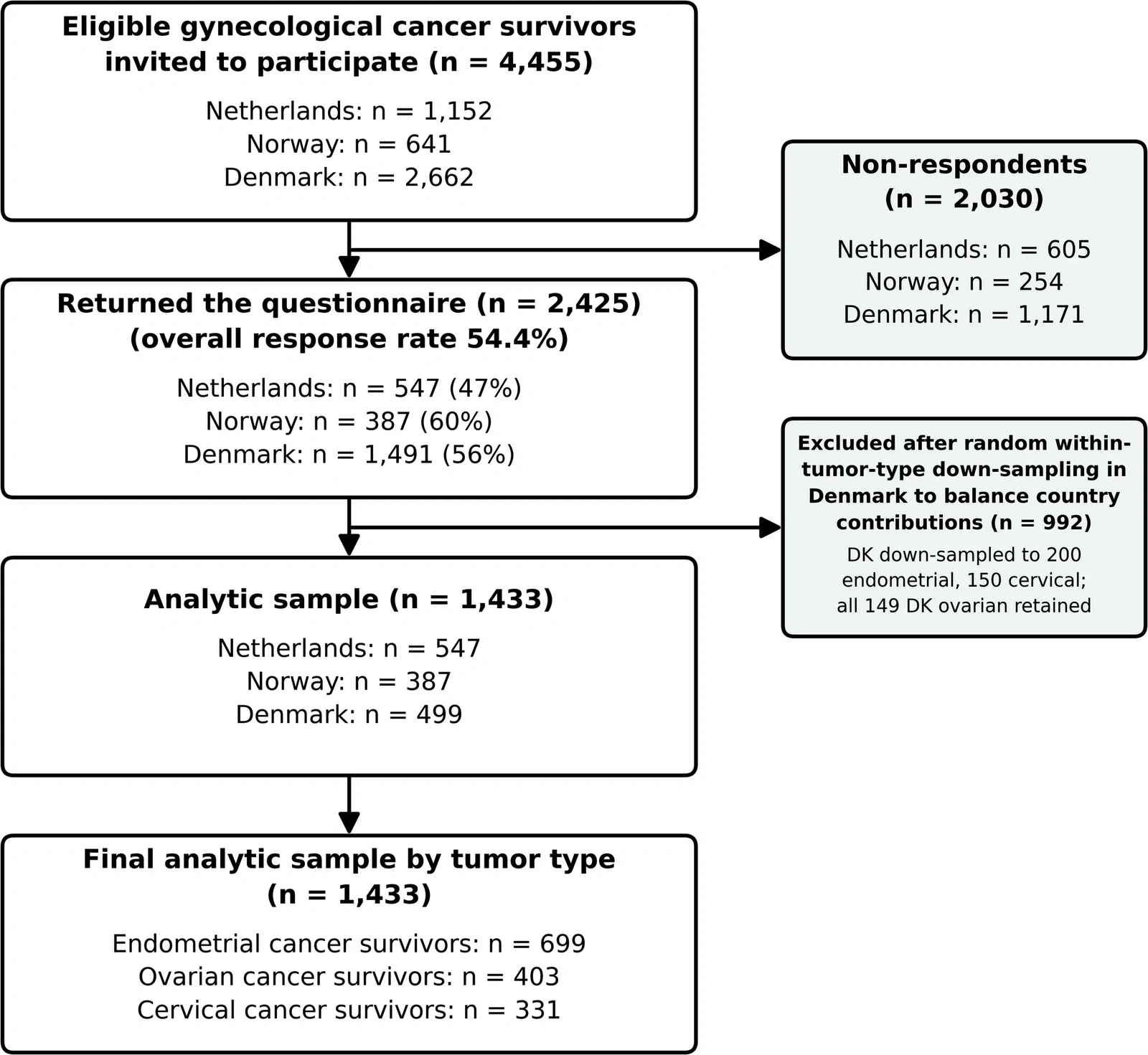
Participant flow through the InCHARGE study. The diagram shows the number of gynecological cancer survivors invited to participate, the number who returned the questionnaire by country with response rates, the country-balancing procedure applied to Danish responders (random within-tumor-type down-sampling for endometrial and cervical survivors; all ovarian responders retained), and the final analytic sample by country and by tumor type. Reasons for non-participation were not collected. All regression analyses used complete-case (listwise) deletion; missing values across HeiQ exposure scales ranged from 0.2% to 1.4%, and all participants had a score on the outcome variable (HeiQ Health Directed Activity scale)

The present analyses were conducted as part of the International Collaboration of Healthcare professionals and Researchers for Gynecologic Cancer Survivors’ Empowerment (InCHARGE) study, a multicenter, cross-sectional questionnaire study of gynecological cancer survivors in the Netherlands, Norway, and Denmark [34, 35]. The reporting follows the STROBE statement for cross-sectional studies. Gynecological cancer survivors who were diagnosed with endometrial, ovarian, or cervical cancer between January 2011 and December 2016 were eligible for participation. Survivors were approached if they were 18 years of age or older at the time of recruitment and were able to read and understand the first language of their respective country. Eligible survivors received an invitation letter that contained information about the study, an informed consent form, and a unique identification number. Non-respondents received one reminder. A participant flow diagram is provided in Fig. 1. All disease stages were included, and the only exclusion criteria were age below 18 years and inability to complete the questionnaire in the first language of the respective country; survivors were therefore not excluded on the basis of disease stage, recurrence, or ongoing treatment.

In the Netherlands, survivors from four hospitals were identified from the Netherlands Cancer Registry. The letter of invitation was sent on behalf of the survivor’s (former) treating physician. In Norway, eligible participants were identified by responsible doctors from six Norwegian departments of gynecology who screened the electronic patient system. The responsible hospital doctors approved contact with eligible individuals, and the study organizers sent the invitations. In the Netherlands and Norway, survivors were provided with a secure link to an online questionnaire, but could request a paper questionnaire if preferred. In Denmark, survivors were identified from the Danish National Patient Registry. Questionnaires were distributed electronically via e-Boks, an encrypted online governmental mailbox that hosts communication between Danish citizens and public services. Data were collected between October 2018 and June 2019.

In the Netherlands (*n* = 1,152), Norway (*n* = 641), and Denmark (*n* = 2,662), a total of 4,455 women were invited to participate and received the questionnaire. The response rate was 47%, 60%, and 56% for the Netherlands, Norway, and Denmark, respectively. Due to a large proportion of Danish participants (1,491 responders compared to 547 in the Netherlands and 387 in Norway), 200 endometrial and 150 cervical cancer survivors from Denmark were randomly selected for inclusion in the final sample to ensure equal distribution across countries. This resulted in the exclusion of 992 Danish responders. All responding Danish ovarian cancer survivors (*n* = 149) were included in the final sample. This balancing procedure was implemented to avoid disproportionate influence of one country in the pooled multinational analyses and to facilitate comparability across countries. The Danish endometrial and cervical cancer survivors were selected at random within tumor type. Accordingly, results should be interpreted as reflecting the balanced multinational cohort. All regression models were adjusted for country.

### Measures

Participant characteristics included age, body mass index (BMI), educational level (primary, secondary, medium, high), gynecological tumor type, cancer stage (FIGO staging), years from diagnosis, partner status (having a partner or not), employment (having a paid job or not), and country. Information about cancer stage was obtained from the Netherlands Cancer Registry, from medical charts in Norway, and from the Danish Gynecological Cancer Database. The exact date of diagnosis was obtained from cancer registries in the Netherlands and Denmark, while the year of diagnosis was self-reported by Norwegian cancer survivors. Data on age, height, weight in kilograms (kg), education, partner status, and employment were self-reported. BMI was calculated as weight in kg divided by height in meters (m)^2^. Education from primary school or lower was categorized as primary, up to 2 years of high school or vocational high school was categorized as secondary, completion of high school was categorized as medium, while education at the college or university level was categorized as high. Gynecological tumor type was determined from the cancer registries and clinical databases used to identify eligible survivors, namely the Netherlands Cancer Registry, the electronic patient records screened by the responsible departments in Norway, and the Danish National Patient Registry, ensuring a standardized classification of endometrial, ovarian, and cervical cancer across countries.

Dimensions of self-management were assessed using the HeiQ version 3 [36], which has been validated in Dutch, Norwegian, and Danish populations [37–39]. The HeiQ contains 40 items. Each item is scored on a Likert-type scale ranging from 1 (strongly disagree) to 4 (strongly agree). The items are divided into eight scales, each containing 4–6 items, and a mean score for the scale is calculated, with no items weighted (Supplementary Table 1). The eight HeiQ scales were designed and validated to represent separate independent questionnaires that could be administered either individually or as a panel of indicators; thus, no global score was developed [40]. A comparative fit index above 0.98 has previously been found for all the HeiQ scales [36]. A Cronbach’s alpha of 0.80 or higher has been reported for seven of the scales and 0.70 for Self-Monitoring and Insight [36]. Five of the scales have previously been validated in a cancer population [41]. A Cronbach’s alpha of 0.75 for Skill and Technique Acquisition, 0.87 for Constructive Attitudes and Approaches, 0.84 for Health Service Navigation, 0.85 for Social Integration and Support, and 0.90 for Emotional Well-being have been reported [41]. Seven of the eight HeiQ scales were assessed as independent variables representing different dimensions of self-management. The scale scores are found by averaging the respective items and are presented on the same four-point scale. Thus, the scale scores are directly comparable.

Self-reported PA participation was assessed by the HeiQ scale Health Directed Activity (Supplementary Table 1). Self-reported assessment was used to ensure feasibility and standardized measurement across the three participating countries in this multinational study. The scale measures engagement in healthy activities, with a focus on physical activities, but also includes behaviors such as relaxing when needed. The Health Directed Activity scale should therefore be understood as a composite indicator of health-directed activity with an emphasis on physical activity, rather than as a measure restricted to physical activity alone. The scale contains four items: “On most days of the week, I do at least one activity to improve my health (e.g., walking, relaxation, exercise)”, “I do at least one type of physical activity every day for at least 30 minutes (e.g., walking, gardening, housework, golf, bowls, dancing, Tai Chi, swimming)”, “On most days of the week, I set aside time for healthy activities (e.g., walking, relaxation, exercise)”, “I walk for exercise, for at least 15 minutes per day, most days of the week”. All participants had a score on the Health Directed Activity scale. The other scales had missing values among 0.2–1.4% of the sample.

### Statistical analyses

Differences in baseline characteristics were assessed between the tumor types using analysis of variance (ANOVA) for continuous variables and the chi-square test for categorical variables. Standardized effect sizes were reported to illustrate the magnitude of the differences in participant characteristics and HeiQ scores across tumor types. Eta-squared was used for continuous variables, Kendall’s tau b for ordinal categorical variables, and Cramer’s V for nominal categorical variables. The effect sizes were reported as negligible, small, medium, or large in accordance with Kotrlik and Williams [42]. Pearson correlations between HeiQ scales were performed and included as supplementary (Supplementary Table 2).

The Health Directed Activity scale has not been previously validated in a cancer population. The scale contains two items specific to time in physical activities, and two items about general health-promoting behaviors. Thus, to investigate whether it was appropriate to include all four items as a measure of PA participation, a confirmatory factor analysis of the scale was conducted in the total sample. Based on the results, the complete scale was included and interpreted as PA participation. To assess associations between the self-management scales and PA participation, a multivariable linear regression was performed for each dimension, adjusted for a-priori selected covariates (age, BMI, educational level, FIGO stage, years from diagnosis, partner status, employment, and country). These covariates were included to control for possible confounders of the relationship between self-management and PA participation. Each scale was assessed without adjusting for the other scales, as they are designed to be measures of independent but related domains. Exploratory models, including multiple HeiQ dimensions simultaneously (i.e., mutually adjusted models), were not tested. All scales were scored from 1 to 4, with higher scores indicating better self-management. Unstandardized regression coefficients with 95% confidence intervals (CI) were reported, representing the magnitude of the associations. Regression analyses were conducted using a complete-case approach (listwise deletion). To explore potential heterogeneity by diagnosis, the regression analyses were also conducted stratified by tumor type (endometrial, ovarian, and cervical cancer), and these results are reported in Supplementary Tables 3 and Supplementary Fig. 1. Analyses were conducted using SPSS 25 (IBM Corp., Released 2017. IBM SPSS Statistics for Windows, Version 25.0. Armonk, NY: IBM Corp.) Because the large Danish sample would otherwise contribute disproportionate weight to the pooled estimates, the country-balancing procedure was applied in addition to adjustment for country. Since the excluded Danish participants were selected at random within tumor type, the included and excluded participants were expected to be comparable. To examine the robustness of the findings to the country composition of the sample, three sensitivity analyses were conducted: the models were estimated with and without adjustment for country, repeated while omitting each country in turn, and additionally adjusted for treatment modality (surgery, chemotherapy, and radiotherapy) (Supplementary Table 4). Multicollinearity was assessed using variance inflation factors, the residuals were inspected for normality and homoscedasticity, and heteroscedasticity-consistent (HC3) standard errors were applied.

## Resu lts

In total, 1,433 cancer survivors were included in the present study, with 38.2% from the Netherlands, 27.0% from Norway, and 34.8% from Denmark (Fig. 1). Descriptive statistics and differences between tumor types are presented in Table 1, while scores on the HeiQ scales and differences between tumor types are presented in Table 2. Pearson correlations between the HeiQ dimensions were generally small-to-moderate, with the largest intercorrelations up to *r* = 0.66 (e.g., Social Integration and Support with Constructive Attitudes and Approaches; Supplementary Table 2).

**Table 1:** Participant characteristics across tumor types, separated for endometrial, ovarian, and cervical cancer survivors

	Cancer type					
	Endometrial *n* = 699	Ovarian *n* = 403	Cervical *n* = 331	All types	Effect size	*p*-values
	Mean (SD)	Mean (SD)	Mean (SD)	Mean (SD)	Eta-squared	
Years from diagnosis	4.1 (2.2)	3.9 (1.6)	3.9 (1.9)	4.0 (2.0)	< 0.01	0.160
Age (years)	67.9 (9.4)	63.8 (12.5)	49.1 (13.4)	62.4 (13.6)	***0.30	< 0.001
BMI (kg/m^2^)	29.3 (6.6)	26.1 (4.9)	26.2 (5.7)	27.7 (6.2)	**0.07	< 0.001

	*n* (%)	*n* (%)	*n* (%)	*n* (%)	Kendall’s tau b	
Stage						
I	564 (80.7)	146 (36.2)	216 (65.3)	926 (64.6)		
II	40 (5.7)	45 (11.2)	74 (22.4)	159 (11.1)		
III	59 (8.4)	153 (38.0)	16 (4.8)	228 (15.9)	**0.20	< 0.001
IV	10 (1.4)	45 (11.2)	9 (2.7)	64 (4.5)		
Missing	26 (3.7)	14 (3.5)	16 (4.8)	56 (3.9)		
Education†						
Primary or lower	119 (17.0)	62 (15.4)	34 (10.3)	215 (15.0)		
Secondary	243 (34.8)	117 (29.0)	85 (25.7)	445 (31.1)		
Medium	206 (29.5)	124 (30.8)	126 (38.1)	456 (31.8)	*0.11	< 0.001
High	126 (18.0)	96 (23.8)	86 (26.0)	308 (21.5)		
Missing	5 (0.7)	4 (1.0)	-	9 (0.6)		

					Cramer’s V	
Partner						
Yes	341 (48.8)	176 (43.7)	130 (39.3)	647 (45.2)		
No	355 (50.8)	224 (55.6)	191 (57.7)	770 (53.7)	0.07	0.030
Missing	3 (0.4)	3 (0.7)	10 (3.0)	16 (1.1)		
Country						
The Netherlands	326 (46.6)	155 (38.5)	66 (19.9)	547 (38.2)		
Norway	173 (24.7)	99 (24.6)	115 (34.7)	387 (27.0)	*0.16	< 0.001
Denmark	200 (28.6)	149 (37.0)	150 (45.3)	499 (34.8)		
Employed						
Yes	187 (26.8)	133 (33.0)	237 (71.6)	557 (38.9)		
No	510 (73.0)	267 (66.3)	91 (27.5)	868 (60.6)	**0.38	< 0.001
Missing	2 (0.3)	3 (0.7)	3 (0.9)	8 (0.6)		

*SD *Standard deviation, *BMI* Body mass index

Effect sizes and *p*-values represent differences between tumor types

Effect sizes are reported as * small, ** medium, and ***large

†Primary or lower – highest complete education being primary school (7-10) or less, Secondary – vocational high school (1–2 years), Medium – high school, High – university or college education

**Table 2:** Scores on the scales from the health education impact questionnaire (HeiQ) were calculated as the average score across the respective items and ranged from 1 to 4

HeiQ scales (Score range 1–4)	Mean (SD)	Mean (SD)	Mean (SD)	Mean (SD)	Eta-squared	
Health Directed Activity	3.05 (0.64)	3.17 (0.62)	2.96 (0.69)	3.06 (0.65)	*0.02	< 0.001
Positive and Active Engagement in Life	3.15 (0.53)	3.23 (0.49)	3.16 (0.51)	3.17 (0.51)	< 0.01	0.044
Skill and Technique Acquisition	3.00 (0.52)	3.02 (0.49)	2.94 (0.55)	2.99 (0.52)	< 0.01	0.101
Constructive Attitudes and Approaches	3.19 (0.55)	3.22 (0.52)	3.16 (0.55)	3.19 (0.54)	< 0.01	0.273
Self-Monitoring and Insight	2.98 (0.42)	3.03 (0.42)	2.93 (0.45)	2.98 (0.43)	*0.01	0.006
Health Service Navigation	3.14 (0.53)	3.24 (0.48)	3.08 (0.53)	3.16 (0.52)	*0.01	< 0.001
Social Integration and Support	3.10 (0.58)	3.18 (0.51)	3.07 (0.56)	3.11 (0.56)	*0.01	0.018
Emotional Well-being	3.11 (0.61)	2.95 (0.60)	2.98 (0.62)	3.04 (0.61)	*0.02	< 0.001

*HeiQ* Health education impact questionnaire, *SD* Standard deviation

Effect sizes and *p*-values represent differences between tumor types

Effect sizes are reported as * small, ** medium, and ***large

Confirmatory factor analysis of the Health Directed Activity scale supported a single-factor solution (Comparative Fit Index [CFI] = 0.992; Root Mean Square Error of Approximation [RMSEA] = 0.071; factor loadings = 0.67–0.82). Internal consistency was acceptable (Cronbach’s α = 0.81). All four items were retained and treated as a composite indicator of PA participation.

**Fig. 2:**
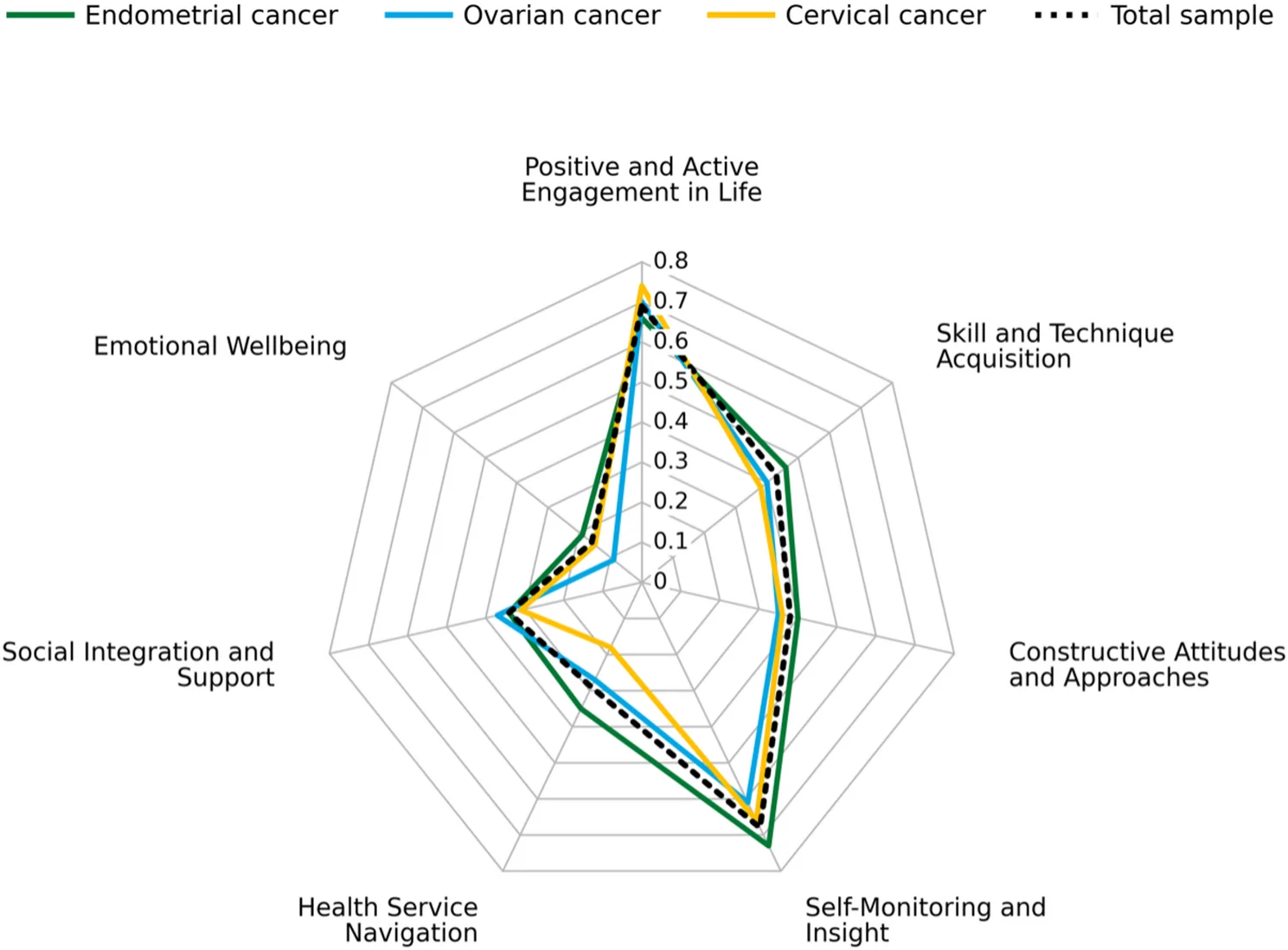
Radar plot of the unstandardized regression coefficients for the associations between self-management scales and physical activity participation in the total sample and among survivors of endometrial, ovarian, and cervical cancer. The regression models were performed for each dimension, adjusted for age, body mass index, educational level, tumor type (in the total sample), cancer stage, years from diagnosis, partner status, employment, and country

In multivariable linear regression models (unstandardized coefficients) adjusted for age, body mass index, educational level, cancer stage, years from diagnosis, partner status, employment, and country, all self-management scales were positively associated with Health Directed Activity, except Emotional Well-being among ovarian cancer survivors (Fig. 2; Supplementary Table 3). The largest associations were observed for Self-Monitoring and Insight and Positive and Active Engagement in Life. For Self-Monitoring and Insight, each 1-point increase corresponded to β = 0.73 (95% CI, 0.63–0.83) in endometrial, 0.61 (95% CI, 0.48–0.75) in ovarian, and 0.67 (95% CI, 0.51–0.82) in cervical cancer survivors. For Positive and Active Engagement in Life, the corresponding estimates were β = 0.66 (95% CI, 0.59–0.74), 0.70 (95% CI, 0.59–0.81), and 0.74 (95% CI, 0.62–0.87), respectively (Fig. 2; Supplementary Table 3). Standardized coefficients for these models are presented in Supplementary Table 3. To aid interpretation, the Health Directed Activity outcome was measured on the same 1 to 4 scale as the self-management exposures, so a regression coefficient of approximately 0.7 indicates that a 1-point higher self-management score was associated with a 0.7-point higher score on the Health Directed Activity scale. Relative to the observed standard deviation of the Health Directed Activity scale (SD = 0.65; Table 2), a difference of this magnitude corresponds to approximately one standard deviation. The associations were robust in sensitivity analyses (Supplementary Table 4). Adjustment for country had a negligible effect on the estimates (for example, Positive and Active Engagement in Life, B = 0.69 both with and without country adjustment; Self-Monitoring and Insight, B = 0.68 versus 0.66), and in analyses omitting each country in turn, these two dimensions remained the most strongly associated with PA participation (Positive and Active Engagement in Life, B = 0.66 to 0.72; Self-Monitoring and Insight, B = 0.63 to 0.72). Additional adjustment for treatment modality did not change the estimates (Positive and Active Engagement in Life, B = 0.69; Self-Monitoring and Insight, B = 0.68). Variance inflation factors indicated no problematic multicollinearity (all below 3.2), and although the residuals showed mild left skew and mild heteroscedasticity, the HC3 standard errors produced consistent conclusions.

## Discussion

The present study aimed to identify the self-management dimensions, as measured by the HeiQ, associated with PA participation among gynecological cancer survivors. Self-management interventions have consistently demonstrated improvements in PA levels in cancer populations [1]. However, evidence regarding feasible and effective approaches to enhance PA levels specifically among gynecological cancer survivors is still lacking. Furthermore, few self-management interventions provide evidence for identifying components effective in enhancing PA behaviors, e.g., by measuring outcomes such as self-efficacy and knowledge [1]. Given the significance of PA in relation to health and well-being, it is critical to target PA behavior through self-management support interventions while clearly reporting intervention components influencing PA behavior change [5].

To the author’s knowledge, no prior study has investigated the associations between self-management skills and PA participation among gynecological cancer survivors. By quantifying which of several distinct self-management dimensions are most strongly associated with PA participation, the present study provides an evidence-based basis for prioritizing candidate intervention targets in a population in which such targets have not previously been identified, and this associational step is a necessary precursor to the design of adequately powered intervention trials. In the present study, strong associations between PA participation and two HeiQ scales: Positive and Active Engagement in Life and Self-Monitoring and Insight were identified. Based on the non-overlapping CIs, Positive and Active Engagement in Life and Self-Monitoring and Insight showed numerically larger associations with PA participation than the other scales among endometrial cancer survivors (Supplementary Fig. 1). However, no formal statistical comparison between the regression coefficients was performed, and these differences should therefore be interpreted descriptively rather than as evidence of significantly stronger associations. Among ovarian and cervical cancer survivors, the Self-Monitoring and Insight scale exhibited overlapping CIs with Skill and Technique Acquisition and Social Integration and Support (ovarian cancer survivors). Positive and Active Engagement in Life did not overlap with scales other than Self-Monitoring and Insight.

The items comprising the Positive and Active Engagement in Life scale measure participants’ ability to convert intention into action and positive outcomes [36]. A high score represents active engagement in life, prioritization of enjoyable and fulfilling hobbies and activities, and motivation to improve life circumstances [36]. The strong association between Positive and Active Engagement in Life and PA participation suggests that activities and hobbies perceived as meaningful life-fulfilling involve some form of PA. This insight can be utilized in self-management interventions, e.g., by exploring enjoyable activities while helping participants plan their schedules. This focus on supporting active engagement is consistent with qualitative findings from gynecological cancer follow-up care, where nurse-led consultations reinforced with eHealth were experienced as facilitating patients’ engagement and self-management in survivorship [43]. Patients with advanced cancer, including ovarian and cervical cancer, have previously reported engaging in healthy behaviors, such as PA, as a means of taking control of their health [44]. Resuming or establishing routines with enjoyable physical activities may contribute to a “normal” and manageable daily routine, facilitating the benefits of PA participation. The association between PA participation and Positive and Active Engagement in Life may reflect autonomous motivation as described by the Self-Determination Theory [29]. Autonomous motivation can involve self-endorsed reasons for engaging in PA, such as finding it enjoyable or feeling in charge. Behaviors originating from personal goals and values indicate autonomy and choice. Interventions emphasizing autonomy by providing participants with choices during PA interventions have shown increased participation and maintenance of PA behaviors [45, 46]. Among women who are overweight, exercise interventions supporting autonomy and informed choices have resulted in higher PA levels and weight loss [47].

A high score on the Self-Monitoring and Insight dimension indicates possession of self-monitoring skills, the ability to take appropriate actions when symptoms worsen, realistic expectations based on health and abilities, and insight into health issues and their influencing factors [36]. This aligns with gynecological cancer follow-up research in which empowerment has been operationalized using the HeiQ Self-Monitoring and Insight domain within a self-management–focused follow-up model (the LETSGO trial), reinforcing monitoring/insight as a clinically relevant survivorship target [48]. The ability to take appropriate actions reflects self-efficacy and perceived competence, which have been associated with positive changes in PA behavior among cancer survivors and are also concepts of the Self-Determination Theory [24, 29, 49]. Relatedly, endometrial cancer survivorship intervention-development work has explicitly emphasized self-regulatory mechanisms (e.g., self-efficacy, knowledge, goal setting, and feedback loops for behavior change) when adapting lifestyle programs targeting PA and sedentary behavior [50]. In endometrial cancer survivors, self-efficacy has also been identified as a key mechanism in PA change over time within an intervention context, supporting the relevance of self-regulatory competence for PA behavior in gynecological survivorship [51]. The ability to find, understand, and use health information has likewise been associated with increased post-treatment PA [52]. As PA is a well-documented means of improving health, this may partly explain the association between the ability to take appropriate action when symptoms worsen and PA participation. Individuals with high Self-Monitoring and Insight, as well as PA participation, may already possess relevant knowledge and healthy behaviors, representing a higher stage of change where health behaviors are performed and maintained [53]. However, addressing a potential discrepancy associated with not adopting appropriate behaviors to improve health may not facilitate behavioral change. Such a discrepancy can create cognitive dissonance, which has been found to predict declines in moderate-to-vigorous PA over time [54]. Targeting cognitive dissonance as a means to change PA behavior has shown inconclusive results and smaller effect sizes compared to using behavioral practice and rehearsal among adults with overweight [55].

An important component of the Self-Monitoring and Insight scale is acknowledging disease-related limitations and adherence to these limits [36]. This reflects individuals who face and accept their condition while being motivated to take the necessary steps to improve it, similar to the concept of resilience. In adult cancer survivors, resilience has been defined as a dynamic process of facing adversity related to the cancer experience, and has been associated with higher levels of PA during cancer treatment [56]. Because resilience and acceptance were not directly measured in this study, these interpretations should be considered hypothetical; future research should include concurrent measures of resilience and/or acceptance to examine whether they map onto the Self-Monitoring and Insight content and how they relate to PA participation.

Although the Self-Determination Theory also encompasses social support and highlights its potential for behavioral change [29], the present study observed a weaker association between Social Integration and Support and PA participation. Previous research indicates that effects on health behavior change, including PA, facilitated by interventions based on the Self-Determination Theory, were mediated by autonomous motivation and perceived competence [32]. This rendered the direct path from intervention to health behavior change non-significant and omitted the social support construct. Skill and Technique Acquisition and Constructive Attitudes and Approaches share some similarities with autonomous motivation and perceived competence as well, but exhibited weaker associations with PA participation. Because these analyses were cross-sectional and did not test indirect pathways, future research should explicitly evaluate mediation models, ideally in longitudinal designs, to examine whether social integration/support and related self-management dimensions influence PA indirectly through constructs such as autonomous motivation and perceived competence.

### Strengths and limitations

The strengths of the present study include the uniquely large sample of gynecological cancer survivors across three countries and the use of a specific, validated, and comprehensive questionnaire for patient education and self-management interventions. A confirmatory factor analysis confirmed the appropriateness of using the complete Health Directed Activity scale as a measure of PA participation. Assessments were conducted separately for gynecological tumor types due to significant differences in cause, symptoms, survival, age at diagnosis, types of treatments typically received, and associated late effects [57].

Some limitations should be considered. First, a cross-sectional design, which cannot determine the impact of behavior (i.e., improved PA levels) or the degree to which a behavior may be changed, was used [58]. Accordingly, the findings should be interpreted as exploratory associations rather than evidence of causality and may reflect bidirectional relationships. In addition, residual confounding is possible; for example, better underlying health status or fewer treatment-related symptoms may contribute to both higher self-management scores and higher PA. Second, the Self-Monitoring and Insight scale has shown the weakest reliability of the HeiQ scales [36]. Third, the sample was likely skewed towards individuals with higher survival rates, as the participants could be several years post-diagnosis. Future longitudinal work should examine whether associations between self-management dimensions and PA differ by survivorship stage (e.g., early vs. later time since diagnosis). Fourth, information on non-participants was not obtained, and the representativeness of the present sample in relation to eligible cancer survivors who declined participation is unknown. As many participants were approached electronically, it is possible that the oldest cancer survivors were not reached or declined participation. Lastly, objective measures of PA were not provided. In addition, the Health Directed Activity scale includes broader health-directed behaviors alongside PA items (e.g., relaxation), and should therefore be interpreted as a composite indicator of health-directed activity with an emphasis on physical activity rather than a pure measure of PA dose. However, the purpose of the present study was not to accurately quantify PA levels but to assess the participants’ desire and intention to participate in PA and whether they reported spending time being physically active during most days. Treatment modality was based on self-report, and although additional adjustment for surgery, chemotherapy, and radiotherapy did not change the associations (Supplementary Table 4), residual confounding by more detailed treatment-related factors, such as dose, timing, and late effects, cannot be fully excluded.

## Conclusions

Across endometrial, ovarian, and cervical cancer survivors, the self-management dimensions Positive and Active Engagement in Life and Self-Monitoring and Insight were consistently and strongly associated with PA participation. The self-management skills of these dimensions may serve as effective components of interventions aimed at promoting PA behaviors.

Further research should assess, preferably in longitudinal and randomized controlled trials, whether interventions targeting these self-management dimensions, for example, by exploring enjoyable activities and helping participants plan their schedules in ways that support regular engagement in health-promoting behaviors, and by incorporating goal setting and structured self-monitoring routines (e.g., tracking PA and reviewing progress to support feedback and problem-solving), can contribute to positive changes in PA levels during gynecological cancer survivorship. Such assessments should be conducted in conjunction with objective monitoring of PA and with measurement of potential mediators (e.g., autonomous motivation and perceived competence) to clarify mechanisms and identify which intervention components drive change. Emerging digital and technology-based approaches, such as eHealth and mobile applications that support self-monitoring, goal setting, and mindfulness- or emotion-focused strategies, may offer scalable ways to target these self-management skills and warrant evaluation in this population, while attention is also given to their limitations, including unequal digital access and variable long-term engagement [43, 59]. 

## Supplementary Information

Supplementary Material 1.

Supplementary Material 2.

## Data Availability

The datasets analyzed during the current study are available from the InCHARGE consortium on reasonable request.

## Abbreviations

PA: Physical activity
HeiQ: Health education impact questionnaire
HDA: Health directed activity
IKNL: Netherlands comprehensive cancer organisation
CoRPS: Center of research on psychological and somatic disorders
REC: Regional committee for medical and health research ethics
SD: Standard deviation
IQR: Interquartile range
CI: Confidence interval
*β*: Unstandardized regression coefficient
n: Number of participants
