# Supplementary material for: Self-management dimensions associated with physical activity participation among gynecological cancer survivors: a cross-sectional study from the international InCHARGE consortium
Source: BMC Womens Health. 2026 Jul 30;26:556. doi: 10.1186/s12905-026-04722-6 (PMC13700420; doi:10.1186/s12905-026-04722-6)
Supplement: Supplementary file 1 [file 12905_2026_4722_MOESM1_ESM.docx]

| **Supplementary Table 1.** The eight self-management scales from the Health Education Impact Questionnaire (HeiQ) (Osborne, Elsworth, & Whitfield, 2007). Each scale is presented with a description of the dimension, one example of an item, and the abbreviation. The Health Directed Activity scale was applied as a measure of self-reported physical activity participation. | | | |
| --- | --- | --- | --- |
| **Dimension** | **Description** | **Example of item (number of items)** | **Abbreviations** |
| Health Directed Activity | High scores indicate high levels of healthful behaviors including exercise | On most days of the week I do at least one activity to improve my health (e.g., walking, relaxation, exercise) (4) | HDA |
| Positive and Active Engagement in Life | High scores characterize someone who is actively engaged in life and is motivated to improve their life circumstances | I am doing interesting things in my life (5) | PAE |
| Skill and Technique Acquisition | High scores are characteristic of someone who has highly developed skills in symptom relief and techniques to manage own health | When I have symptoms, I have the skills that help me cope (4) | STA |
| Constructive Attitudes and Approaches | High scores identify individuals who attempt to minimize the effects of illness and are determined not to allow the illness to control their life | I try not to let my health problems stop me from enjoying life (5) | CAA |
| Self-Monitoring and Insight | High scores identify self-monitoring, self-management, setting reasonable limits or targets, and insight into living with a health problem | When I have health problems, I have a clear understanding of what I need to do to control them (6) | SMI |
| Health Services Navigation | High scores characterize a person who is confident in their ability to communicate with healthcare professionals and has good understanding of ways to access healthcare in order to get their needs met | I communicate very confidently with my doctor about my healthcare needs (5) | HSN |
| Social Integration and Support | High scores indicate high levels of social interaction, high sense of support, seeking support from others, and low levels or social isolation due to illness | Overall, I feel well looked after by friends and family (5) | SIS |
| Emotional Wellbeing (reversed scale) | Low scores identify individuals who have high levels of overall health-related negative affect, negative attitude to life, and high levels of anxiety, stress, anger and depression | I get upset when I think about my health (6) | EW |

**Supplementary Table 2.** Pearson correlations between all HeiQ scales.

|  | HDA | PAE | EW | SMI | CAA | STA | SIS |  |  |
| --- | --- | --- | --- | --- | --- | --- | --- | --- | --- |
| PAE | .559** |  |  |  |  |  |  |  |  |
| EW | .170** | .366** |  |  |  |  |  |  | >.600 |
| SMI | .464** | .529** | .134** |  |  |  |  |  | >.500 |
| CAA | .342** | .610** | .531** | .392** |  |  |  |  | >.400 |
| STA | .363** | .553** | .411** | .552** | .619** |  |  |  | >.300 |
| SIS | .324** | .519** | .389** | .389** | .664** | .593** |  |  | >.200 |
| HSN | .266** | .437** | .298** | .402** | .562** | .574** | .603** |  | >.100 |

**p<0.001

HDA – Health Directed Activity; PAE – Positive and Active Engagement in Life; EW – Emotional Wellbeing; SMI – Self-Monitoring and Insight; CAA – Constructive Attitudes and Approaches; Skill and Technique Acquisition; SIS- Social Integration and Support; HSN – Health Service Navigation.

**Supplementary Table 3.** All coefficients, confidence intervals, and standardized coefficients from the adjusted linear regression performed between Health Directed Activity and the other self-management scales from the Health Education Impact Questionnaire, in the total sample and within each tumor type.

|  | PAE | STA | CAA | SMI | HSN | SIS | EW |
| --- | --- | --- | --- | --- | --- | --- | --- |
| **Overall** |  |  |  |  |  |  |  |
| Regression coefficient | .69 | .43 | .38 | .68 | .29 | .34 | .16 |
| CI | .64, .75 | .36, .49 | .32, .44 | .61, .75 | .22, .35 | .28, .40 | .10, .21 |
| p-value | <.001 | <.001 | <.001 | <.001 | <.001 | <.001 | <.001 |
| Standardized coefficient | .55 | .34 | .32 | .45 | .23 | .29 | .15 |
| **Endometrial cancer** |  |  |  |  |  |  |  |
| Regression coefficient | .66 | .46 | .40 | .73 | .35 | .34 | .19 |
| CI | .59, .74 | .37, .54 | .32, .48 | .63, .83 | .26, .44 | .26, .42 | .11, .27 |
| p-value | <.001 | <.001 | <.001 | <.001 | <.001 | <.001 | <.001 |
| Standardized coefficient | .55 | .38 | .35 | .48 | .29 | .32 | .18 |
| **Ovarian cancer** |  |  |  |  |  |  |  |
| Regression coefficient | .70 | .40 | .35 | .61 | .27 | .37 | .09 |
| CI | .59, .81 | .28, .53 | .23, .47 | .48, .75 | .14, .41 | .25, .50 | -.02, .20 |
| p-value | <.001 | <.001 | <.001 | <.001 | <.001 | <.001 | .104 |
| Standardized coefficient | .55 | .31 | .29 | .42 | .21 | .30 | .09 |
| **Cervical cancer** |  |  |  |  |  |  |  |
| Regression coefficient | .74 | .38 | .36 | .66 | .18 | .31 | .15 |
| CI | .62, .87 | .25, .51 | .23, .50 | .51, .82 | .04, .32 | .18, .44 | .01, .28 |
| p-value | <.001 | <.001 | <.001 | <.001 | .015 | <.001 | .031 |
| Standardized coefficient | .56 | .31 | .30 | .44 | .14 | .25 | .13 |

PAE – Positive and Active Engagement in Life; EW – Emotional Wellbeing; SMI – Self-Monitoring and Insight; CAA – Constructive Attitudes and Approaches; Skill and Technique Acquisition; SIS- Social Integration and Support; HSN – Health Service Navigation; CI – confidence interval.

PAE – Positive and Active Engagement in Life; EW – Emotional Wellbeing; SMI – Self-Monitoring and Insight; CAA – Constructive Attitudes and Approaches; Skill and Technique Acquisition; SIS- Social Integration and Support; HSN – Health Service Navigation

**Supplementary Figure 1.** The regression coefficients with confidence intervals for the associations between self-management scales and physical activity participation within each tumor type.

**Supplementary Table 4. Sensitivity analyses for the associations between the self-management scales and physical activity participation (Health Directed Activity) in the total sample. Values are unstandardized regression coefficients (B) with 95% confidence intervals. Unless stated otherwise, models were adjusted for age, body mass index, educational level, tumor type, cancer stage, years from diagnosis, partner status, employment, and country.**

| **Self-management scale** | **Main model** | **Without country adjustment** | **With treatment adjustment** | **Excluding the Netherlands** | **Excluding Norway** | **Excluding Denmark** |
| --- | --- | --- | --- | --- | --- | --- |
| Positive and Active Engagement in Life | 0.69 (0.64, 0.75) | 0.69 (0.63, 0.74) | 0.69 (0.64, 0.75) | 0.69 (0.62, 0.76) | 0.66 (0.60, 0.73) | 0.72 (0.66, 0.79) |
| Self-Monitoring and Insight | 0.68 (0.61, 0.75) | 0.66 (0.59, 0.73) | 0.68 (0.61, 0.75) | 0.70 (0.61, 0.79) | 0.63 (0.55, 0.71) | 0.72 (0.63, 0.82) |
| Skill and Technique Acquisition | 0.43 (0.36, 0.49) | 0.43 (0.36, 0.49) | 0.43 (0.36, 0.49) | 0.40 (0.32, 0.47) | 0.40 (0.33, 0.47) | 0.49 (0.41, 0.57) |
| Constructive Attitudes and Approaches | 0.38 (0.32, 0.44) | 0.38 (0.32, 0.44) | 0.38 (0.32, 0.44) | 0.36 (0.29, 0.44) | 0.35 (0.28, 0.43) | 0.42 (0.35, 0.49) |
| Social Integration and Support | 0.34 (0.28, 0.40) | 0.34 (0.28, 0.40) | 0.34 (0.28, 0.40) | 0.32 (0.24, 0.40) | 0.32 (0.25, 0.39) | 0.38 (0.31, 0.46) |
| Health Service Navigation | 0.29 (0.22, 0.35) | 0.29 (0.22, 0.35) | 0.29 (0.22, 0.35) | 0.26 (0.18, 0.34) | 0.28 (0.20, 0.35) | 0.33 (0.25, 0.41) |
| Emotional Well-being | 0.16 (0.10, 0.21) | 0.16 (0.10, 0.22) | 0.16 (0.10, 0.21) | 0.14 (0.06, 0.21) | 0.14 (0.08, 0.21) | 0.20 (0.13, 0.27) |

*Main model: the primary adjusted model. Without country adjustment: country removed from the model. With treatment adjustment: additionally adjusted for surgery, chemotherapy, and radiotherapy. Excluding a country: the model re-estimated after omitting that country. Across all models, Positive and Active Engagement in Life and Self-Monitoring and Insight remained the self-management dimensions most strongly associated with physical activity participation. B – unstandardized regression coefficient; CI – confidence interval.*

# References

Osborne, R. H., Elsworth, G. R., & Whitfield, K. (2007). The Health Education Impact Questionnaire (heiQ): An outcomes and evaluation measure for patient education and self-management interventions for people with chronic conditions. *Patient Education and Counseling, 66*, 192-201.
