# Supplementary material for: Self-management dimensions associated with physical activity participation among gynecological cancer survivors: a cross-sectional study from the international InCHARGE consortium
Source: BMC Womens Health. 2026 Jul 30;26:556. doi: 10.1186/s12905-026-04722-6 (PMC13700420; doi:10.1186/s12905-026-04722-6)
Supplement: Supplementary file 2 [file 12905_2026_4722_MOESM2_ESM.docx]

STROBE Statement—Checklist of items that should be included in reports of ***cross-sectional studies***

|  | Item No | Recommendation | Page/Section No |
| --- | --- | --- | --- |
| **Title and abstract** | 1 | (*a*) Indicate the study’s design with a commonly used term in the title or the abstract | p. 1; p. 2 |
|  |  | (*b*) Provide in the abstract an informative and balanced summary of what was done and what was found | p. 2 |
| Introduction | | |  |
| Background/rationale | 2 | Explain the scientific background and rationale for the investigation being reported | pp. 3–5 |
| Objectives | 3 | State specific objectives, including any prespecified hypotheses | p. 5 |
| Methods | | |  |
| Study design | 4 | Present key elements of study design early in the paper | p. 5 |
| Setting | 5 | Describe the setting, locations, and relevant dates, including periods of recruitment, exposure, follow-up, and data collection | pp. 5–6 |
| Participants | 6 | (*a*) Give the eligibility criteria, and the sources and methods of selection of participants | pp. 5–6 |
| Variables | 7 | Clearly define all outcomes, exposures, predictors, potential confounders, and effect modifiers. Give diagnostic criteria, if applicable | pp. 7–8; Suppl. Table 1 |
| Data sources/ measurement | 8* | For each variable of interest, give sources of data and details of methods of assessment (measurement). Describe comparability of assessment methods if there is more than one group | pp. 6–8; Suppl. Table 1 |
| Bias | 9 | Describe any efforts to address potential sources of bias | p. 6; p. 10; pp. 20–21 |
| Study size | 10 | Explain how the study size was arrived at | pp. 6, 10 |
| Quantitative variables | 11 | Explain how quantitative variables were handled in the analyses. If applicable, describe which groupings were chosen and why | pp. 7–8 |
| Statistical methods | 12 | (*a*) Describe all statistical methods, including those used to control for confounding | pp. 9–10 |
|  |  | (*b*) Describe any methods used to examine subgroups and interactions | p. 10; pp. 14–15; Suppl. Table 3; Suppl. Figure 1 |
|  |  | (*c*) Explain how missing data were addressed | pp. 9–10 |
|  |  | (*d*) If applicable, describe analytical methods taking account of sampling strategy | pp. 6, 9 |
|  |  | (*e*) Describe any sensitivity analyses | pp. 10, 14–15; Suppl. Table 4 |
| Results | | |  |
| Participants | 13* | (a) Report numbers of individuals at each stage of study—eg numbers potentially eligible, examined for eligibility, confirmed eligible, included in the study, completing follow-up, and analysed | pp. 6, 10–11 |
|  |  | (b) Give reasons for non-participation at each stage | p. 21 |
|  |  | (c) Consider use of a flow diagram | p. 11 |
| Descriptive data | 14* | (a) Give characteristics of study participants (eg demographic, clinical, social) and information on exposures and potential confounders | pp. 12–13 |
|  |  | (b) Indicate number of participants with missing data for each variable of interest | p. 9; pp. 12–13 |
| Outcome data | 15* | Report numbers of outcome events or summary measures | pp. 13–14 |
| Main results | 16 | (*a*) Give unadjusted estimates and, if applicable, confounder-adjusted estimates and their precision (eg, 95% confidence interval). Make clear which confounders were adjusted for and why they were included | pp. 14–15; Suppl. Table 3 |
|  |  | (*b*) Report category boundaries when continuous variables were categorized | pp. 7–8 |
|  |  | (*c*) If relevant, consider translating estimates of relative risk into absolute risk for a meaningful time period | Not applicable |
| Other analyses | 17 | Report other analyses done—eg analyses of subgroups and interactions, and sensitivity analyses | pp. 14–15; Suppl. Table 2; Suppl. Table 3; Suppl. Table 4; Suppl. Figure 1 |
| Discussion | | |  |
| Key results | 18 | Summarise key results with reference to study objectives | p. 16; pp. 21–22 |
| Limitations | 19 | Discuss limitations of the study, taking into account sources of potential bias or imprecision. Discuss both direction and magnitude of any potential bias | pp. 20–21 |
| Interpretation | 20 | Give a cautious overall interpretation of results considering objectives, limitations, multiplicity of analyses, results from similar studies, and other relevant evidence | pp. 16–22 |
| Generalisability | 21 | Discuss the generalisability (external validity) of the study results | p. 21 |
| Other information | | |  |
| Funding | 22 | Give the source of funding and the role of the funders for the present study and, if applicable, for the original study on which the present article is based | p. 23 |
